# Proximity of breeding and foraging areas affects foraging effort of a crepuscular, insectivorous bird

**DOI:** 10.1038/s41598-018-21321-0

**Published:** 2018-02-14

**Authors:** Ruben Evens, Natalie Beenaerts, Thomas Neyens, Nele Witters, Karen Smeets, Tom Artois

**Affiliations:** 1 Hasselt University, Centre for Environmental Sciences, Research Group: Zoology, Biodiversity and Toxicology, Campus Diepenbeek, Agoralaan, Gebouw D, 3590 Diepenbeek, Belgium; 2 Hasselt University, Centre for Statistics, Research Group: I-BIOSTAT, Campus Diepenbeek, Agoralaan, Gebouw D, 3590 Diepenbeek, Belgium; 3 Hasselt University, Centre for Environmental Sciences, Research Group: Environmental Economics, Campus Diepenbeek, Agoralaan, Gebouw D, 3590 Diepenbeek, Belgium

## Abstract

When complementary resources are required for an optimal life cycle, most animals need to move between different habitats. However, the level of connectivity between resources can vary and, hence, influence individuals’ behaviour. We show that landscape composition and configuration affect the connectivity between breeding (heathlands) and foraging habitats (extensively-grazed grasslands) of the European Nightjar (Caprimulgus europaeus), a crepuscular insectivorous bird. On a daily basis, nightjars connect breeding and foraging sites by rapidly crossing unsuitable habitats in order to exploit a higher prey biomass in foraging sites. However, low availability of foraging habitat near breeding sites and clustered landscapes greatly increase foraging distance. Birds occupying these sub-optimal breeding areas compensate for longer travels by increasing foraging duration, and their physiology shows increased stress levels. All findings suggest that landscape heterogeneity can affect population dynamics of nightjars. Therefore, we recommend an integrated management approach for this EU-protected bird species.

## Introduction

Movement is essential for almost all animals on earth and influences processes ranging from the individual to the ecosystem level^1,2^. In order to link vital resources, such as nesting and feeding sites, many species undertake daily movements between complementary habitats^3–5^. However, the quality of, and accessibility to these resources can vary^6–10^.

Human pressures on natural habitats cause rapid changes in landscape composition and configuration^11,12^. Recent studies have shown that some species, such as Red-necked Nightjars *Caprimulgus ruficollis* and Marsh Harrier *Circus aeruginosus*, benefit from anthropogenic landscape modification^13,14^. However, more often, these modifications reduce the availability of resources and/or decrease the connectivity between complementary habitats^15^. Consequently, bird species can experience increased energy expenditure^16^, lower foraging efficiency^6,17^, higher predation risk^18^ and increased physiological costs^19^, which all can lead to changes in population dynamics^20,21^. Understanding how animals move in heterogeneous environments is, therefore, critical in the framework of the conservation of species and the sustainable management of altered landscapes^1,2,12,22^.

The European Nightjar *Caprimulgus europaeus*, a crepuscular, insectivorous, EU-protected bird species (European Birds Directive 2009/147/EC) uses complementary habitats within its home range for different resources^5^. In Flanders (Northern Belgium), breeding areas of this sub-Saharan migratory bird are mainly found on open semi-natural, low-nutrient habitats, such as heathlands and inland dunes (hereafter together referred to as heathlands)^23^. Whereas nightjars rely on their camouflage to stay well-hidden from predators during daytime, at night they use complementary habitats, such as extensively-cultivated grasslands and wet grasslands, to forage for aerial insects^5^. The nightjar’s typical breeding habitat has decreased by 95% due to anthropogenic activities over the last 150 years^24^. Consequently, breeding habitats now only occur in fragmented landscapes dominated by woodland, agricultural, urban and industrial areas.

There is ample evidence for many animal groups, including nightjars^5^, that home range size and foraging distance are influenced by habitat composition^21,25–28^. When foraging distances increase and animals invest more time in foraging their fitness can reduce^6,16,20,28,29^ which could be reflected in biomarkers of oxidative stress^30–32^. In this study, we aim to further disentangle the foraging behaviour of nightjars in Flanders, using GPS technology, refined habitat maps and measures of plasma anti-oxidants. We tested whether 1) both measures of landscape heterogeneity: i.e. habitat composition, and habitat configuration, influence nightjars’ foraging distance (see sections on “*Landscape heterogeneity*”); 2) landscape heterogeneity affects nightjars’ foraging behaviour (i.e. foraging distance, foraging time and habitat specific travel speed; see sections on “*Foraging ecology*”); 3) nightjars’ specific choice of foraging habitat could be explained by habitat-dependant food availability patterns (see sections on “*Foraging economy*”) and 4) landscape heterogeneity could induce increased stress levels when birds occupy sub-optimal breeding areas (see sections on “*Landscape economy*”).

## Methods

**Figure 1:**
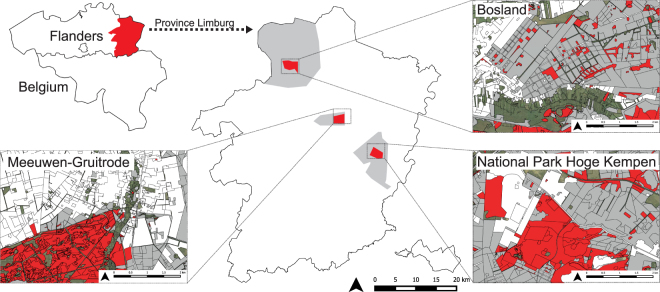
Our study areas (central map: red areas) situated in the Province of Limburg in Flanders (Belgium), more specifically they are situated in Bosland, the military area of Meeuwen-Gruitrode and the Mechelse Heide in National Park Hoge Kempen (central map: grey areas). These study areas are different as to their configuration and composition of functional habitats (same colour code as in Fig. 2): breeding (red), foraging (dark-grey), roosting (grey) and unsuitable habitats (white). The smaller habitat maps were modified from the Biological Value Map V2.0 under open data access of INBO; maps were created using QGIS 2.12 Lyon (Open Source Geospatial Foundation Project, http://qgis.osgeo.org) and edited using Adobe Illustrator CC www.adobe.com.

**Figure 2:**
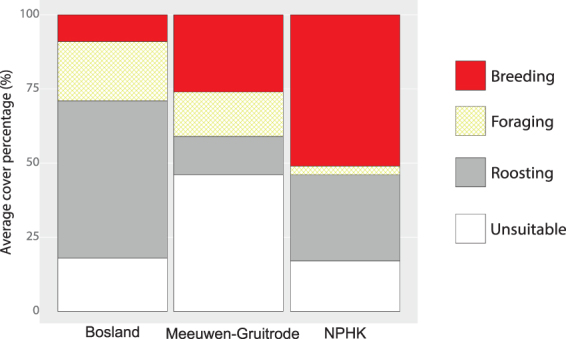
Average cover percentage of functional habitats in the three study areas: Bosland, the military area of Meeuwen-Gruitrode and the Mechelse Heide in National Park Hoge Kempen (NPHK). Habitat composition was calculated from the amount of habitat that was available for each initial foraging flight (average foraging distance).

GPS-tracking was conducted from May to August during three consecutive years (2014–2016) at three sites in Flanders, Belgium: Bosland (51.17°N, 5.34°E), the military area Meeuwen-Gruitrode (51.04°N, 5.30°E) and the Mechelse Heide in National Park Hoge-Kempen (NPHK; 50.98°N, 5.63°E; Fig. 1). The sites are located approximately 15 km apart (Fig. 1) and were selected because they are different as to their configuration and composition of functional habitats (Fig. 2). Functional habitats are described following the criteria of Evens *et al*.^5^: 1) breeding habitat (dry heathland, inland dunes and forest clearings); 2) roosting habitats (pine stands); 3) foraging habitat (extensively-cultivated farmlands, oak and poplar stands, recreational areas, wet heathlands, swamps and riverine valleys); and 4) unsuitable habitat (urbanised areas, intensively-cultivated farmlands and anthropogenic water bodies). Most breeding habitats are included as Special Protected Areas in Natura 2000 (75–80% Bosland, 100% Meeuwen-Gruitrode and 100% Mechelse Heide). The study sites are different as to their configuration of breeding habitats (fragmented vs. continuous) and as to area of unsuitable habitats that separate breeding sites from foraging sites (high vs. low; Fig. 2). The centre of Bosland consists of a mosaic landscape (2500 ha) with coniferous trees (82%), deciduous trees (14%) and fragmented heathlands (4%). In Bosland, large areas with potential foraging habitat are located in proximity to breeding areas^5^. The military area of Meeuwen-Gruitrode and the Mechelse Heide hold two of the most important continuous heathlands in Flanders (circa 1000 ha and 800 ha respectively). Potential foraging habitats in Meeuwen-Gruitrode are found adjacent to the breeding habitats and consist of remnant fragments within agricultural land. Larger areas with potential foraging sites are found in the Mechelse Heide, but pine forests, industrial areas and cities separate them from breeding sites.

During our study we captured 48 nightjars (Bosland: 29; Meeuwen-Gruitrode: 8; NPHK: 11; Supplementary Table T1) using ultra fine mist-nets (Ecotone, 12 × 3m) and tape lures. All birds were captured within their territories and marked with a unique alphanumeric ring from the Royal Belgian Institute of Natural Sciences (RBINS). We noted individual information such as sex (male or female), age (older than 1st calendar year (CY), 2nd CY, or > 2CYs), weight (to the nearest 0.1 gram) and wing length (measured to the nearest millimetre). We fitted a Pathtrack Ltd. nanoFix or Biotrack Ltd. PinPoint-40 GPS-logger dorsally between the wings with a full body harness made of Teflon ribbon (Bally Ribbon)^33^. Tags weighed less than 3% of the average weight of tagged birds (72.10 ± 8.68 g; for a list of tagged birds see Supplementary Table T1). GPS-tags were programmed to start logging 24-hours after deployment, from dusk until dawn. Biotrack Ltd. PinPoint V1 loggers were programmed to log a total of 40 locations, once every 15 minutes, in one night. The other tags were able to log a bird’s movement from three up to six nights, depending on the sampling interval (3 or 4 minutes for Biotrack Ltd. PinPoint-40 V2 and Pathtrack nanoFix V1 & V2) and the type of vegetation cover, as shown by Forin-Wiart *et al*.^34^. We performed attempts to recapture the birds one week after deployment. Upon successful recapture, tracking data were downloaded and imported in Quantum GIS V2.12. Environmental data were derived from nearest (approximately ten kilometres) online weather stations in Hechtel (Bosland), Bree (Meeuwen-Gruitrode) and Maaseik (NPHK).

### Foraging ecology

We followed seven steps to calculate foraging distance, flight speed and foraging time (see Supplementary Methods M1 for full details of these calculations). For each night and each bird we defined foraging events with *initial foraging flights* as a measure of foraging distance. Initial foraging flights were defined as the first flights of the night, starting from breeding/roosting sites towards the foraging habitats and subsequent flights performed by birds that were present for at least one hour at the breeding/roosting site after they performed previous foraging flights. We defined complete foraging tracks (see below) as movements that include the start at breeding/roosting site, flight towards foraging habitats, foraging, return flight to breeding site and arrival at breeding/roosting site. An individual can perform more than one complete foraging track per night.

### Landscape heterogeneity

We created a tailored structural and functional habitat map^12^ per study site. The structural landscape map is based on the Biological Value Map of Flanders^35^ and we reclassified 7500 habitat types into 23 relevant habitat types, following the criteria of Evens *et al*.^5^. We then created the functional landscape map by grouping the 23 habitat types into four functional categories following the criteria of Evens *et al*.^5^.

For each foraging event (see above), we cut out four circular sub-maps from the structural and the functional habitat map, to delineate the area of available habitats and to derive measures of local landscape heterogeneity related to that foraging event^5^ (Supplementary Methods M2). The centre of each circle was placed at the start position of the corresponding foraging event. In other words, we created two sub-maps (one from the structural and one from the functional habitat map) with a radius equal to the initial foraging distance and two sub-maps (one from the structural and one from the functional habitat map) with a radius equal to the average foraging distance (1650 m; calculated as the average foraging distance of all initial foraging flights for all individuals).

Finally, from the sub-maps, we quantified the percentage of available foraging habitat and habitat diversity (Shannon diversity index) as measures of habitat composition. To measure habitat configuration we classified the spatial arrangement of functional habitats into three categories using Moran’s I^36^. Moran’s I measures spatial autocorrelation and indicates whether the configuration of habitat types is 1) random (p ≥ 0.05) or non-random (p ≤ 0.05), with similar habitat types to be 2) clustered (z-score ≥ 1.96) or 3) dispersed (z-score ≤ −1.96). Furthermore, we calculated the average patch size of foraging habitats as a second measure of habitat configuration. We used the sub-maps with available functional habitats (average foraging distance) to calculate the average rea of each functional habitat type for each study area (Fig. 2).

### Foraging economy

We quantified the abundance of nocturnal insects in breeding, foraging and roosting habitats during three consecutive years (2011–2014, May-August) in Bosland. At least three times per week we used eight insect traps with 15watt UVA-lamps (attraction radius for photosensitive insects is approximately five meters^37^) to catch nocturnal insects from dusk until dawn. Insects were trapped in a mixture of 70% ethanol, 30% water and a drop of detergent. In this study we focussed on moths (*Lepidoptera*), as these constitute the main diet of nightjars^38^. Specimens of moths were removed from the liquid at dawn, dried and wing lengths were measured to the nearest millimetre. To estimate biomass, we used wing length as a measure, as proposed by Garcia-Barros^39^. Other insects were picked out and have been preserved for other, general biodiversity studies.

We collected blood samples from adult nightjars in all study sites during the breeding season of 2016. A maximum of 300 µl blood was extracted from the brachial vein a few minutes after the bird was captured. The blood was immediately centrifuged in the field and the plasma and red-blood cells were stored separately in liquid nitrogen. Within 24-hours the samples were stored at −80 °C until laboratory analysis, which occurred within five months after sampling. We used the –SHp test (Diacron International) to quantify the plasma concentration of total thiols (e.g. albumin, lipoic acid and glutathione, expressed as micromolar of –SH groups), a biomarker for an individual’s fitness^31,32^.

All methods were carried out in accordance with relevant guidelines and regulations as licenced by the Royal Belgian Institute for Natural Sciences and the Flemish Agency for Nature and Forest.

### Statistical analysis

#### Landscape heterogeneity

To investigate the effect of individual, environmental and landscape characteristics on foraging distance we fitted linear mixed models to our data (LMM^40^). Model selection was based on a backwards selection procedure. Due to multicollinearity issues the selection process was initiated from two separate models. More specific, correlation was found between the following environmental variables (for more details concerning the variables, see below): the amount of available foraging habitat and Moran’s I, the amount of available foraging habitat and the average size of foraging sites, habitat diversity and Moran’s I, and habitat diversity and the size of foraging sites. Before variable elimination, model 1 contained foraging distance (log-transformed) as outcome variable, and individual (sex [M/F], age [≥1CY, 2CY, ≥2CYs], wing length [mm]), environmental data (maximal daily temperature [°C], total daily rainfall [mm], moon phase^41^ [% moon face illuminated]), habitat characteristics (amount of foraging habitat [calculated using the average foraging distance sub-map; % cover], habitat diversity [calculated using the initial foraging distance sub-map; Shannon Index]) and year [20014, 2015, 2016] as fixed effects. We introduced a random intercept to correct for correlation between observations within individuals, which was nested in location [Bosland, Meeuwen-Gruitrode, NPHK] to account for unexplained variation between research areas. A second version of model 1 was also fitted containing available foraging habitat (initial foraging distance instead of average foraging distance; % cover). In model 2, the variable selection process was initiated from a full model with, again, foraging distance (log-transformed) as outcome and a random intercept per individual, nested within location, but with the fixed effects now being individual (sex [M/F], age [≥1CY, 2CY, ≥2CYs], wing length [mm]), environmental (maximal daily temperature [°C], total daily rainfall [mm], daily moon phase [% moon face illuminated]) and different habitat characteristics (Moran’s I [calculated using the initial foraging distance sub-map; classified as random, dispersed or clustered]) and average size of foraging habitats [calculated using the initial foraging distance sub-map; m²]).

#### Foraging ecology

Using LMMs, we also assessed possible differences between the three study sites regarding foraging distance (log-transformed; random effect: individual) and the area of suitable foraging habitat in proximity to breeding/roosting sites (square root-transformed; average foraging distance; random effect: individual). The effect of foraging distance on foraging duration (log-transformed) was analysed using an LMM (random effect: individual nested within location). Similarly, habitat-specific flight speeds were compared between unsuitable (locations with dense forest, agricultural and urban area) and suitable habitats (locations with heathland and grassland; binary explanatory variable: habitat suitability, random effect: individual).

#### Foraging economy

To evaluate the influence of habitat type and temperature on the observed insect biomass (log-transformed), another LMM was used (fixed effects: habitat type, temperature and year; random effect: trap number). Finally, we performed an ANOVA to assess whether the plasma concentration of thiols differed between the three research areas.

For all LMM’s approximate F-tests were used to assess significance. Model assumptions for both LMM and ANOVA analysis were thoroughly checked, via plots of marginal and conditional residuals against fitted values to assess homoscedasticity and linearity. QQ plots of both types of residuals were used to evaluate normality. Correlation tests and variance inflation factor (VIF) investigations were used to detect problems with multicollinearity^40^. Post-hoc pairwise comparison p-values were adjusted via the Tukey-Kramer method to correct for inflation of Type I errors caused by multiple testing^42^. Lastly, for all hypothesis tests a significance level of 5% was used.

The datasets generated during the current study are available from the corresponding author on reasonable request.

## Results

**Figure 3:**
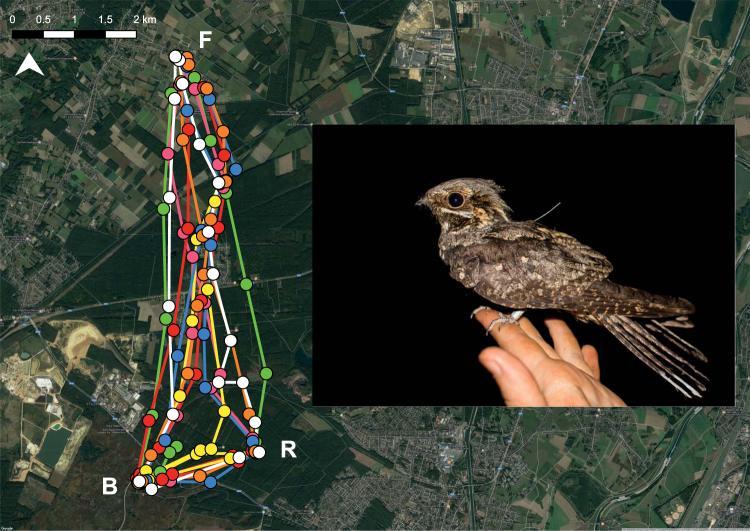
Foraging behaviour of one nightjar that was tracked for seven nights in NPHK. Each colour represents tracking data of one night. Each point is one GPS position and lines connect subsequent GPS positions. R = roosting site in pine forest, F = foraging site in extensively-grazed grassland, B = breeding site in heathland. Embedded photo shows a nightjar carrying a GPS-logger. The background map was under open data access of Google Maps; tracking data was plotted using QGIS 2.12 Lyon (Open Source Geospatial Foundation Project, http://qgis.osgeo.org) and edited using Adobe Illustrator CC www.adobe.com.

We recaptured 31 nightjars carrying GPS-loggers and 30 loggers (Fig. 3) contained data on foraging behaviour (Bosland: 20; Meeuwen-Gruitrode: 4; NPHK: 6; Supplementary Table T1). We collected 16385 GPS-observations (30 individuals; 829 ± 298 observations; max = 1145, min = 40) comprising 463 flight paths. We identified 133 complete foraging tracks, 210 initial foraging flights (Fig. 3), compared foraging duration for 160 foraging flights and examined habitat-specific flight speed for 199 flight observations. We collected and analysed 58 plasma samples of adult nightjars and analysed food availability using 448 samples. The results of the statistical analyses are summarized in Table 1.

**Table 1:** Summary of the results from the statistical analyses. For each analysis we show the outcome variables, random and fixed effects. *F test in ANOVA and an approximate F test in LMM, **Tukey-adjusted p-values, Foraging habitat^1^ = average foraging distance, Foraging habitat² = initial foraging distance.

Analysis	Model	N	Outcome	Random Effects		Fixed Effects						Posthoc Tests			
				Variable	Estimation	Variable	estimation	dfN	dfD	F*	p-value	Difference	Estimate (transformed)	t	p-value**
1	LMM	210	ln(Foraging distance)	Location	0.4497 (0.4921)	Foraging habitat^1^	−2.5365 (0.5338)	1	203	22.58	<0.0001				
				Bird ID (Location)	0.1116 (0.0385)	Habitat diversity	1.7135 (0.0987)	1	198	301.54	<0.0001				
2	LMM	210	ln(Foraging distance)	Location	0.6999 (0.7472)	Foraging habitat²	−0.09272 (0.2872)	1	197	0.10	<0.7471				
				Bird ID (Location)	0.1528 (0.0498)	Habitat diversity	1.6144 (0.1029)	1	196	246.31	<0.0001				
3	LMM	210	ln(Foraging distance)	Location	0.5555 (0.6305)	Size foraging habitats	0.000012 (4.399E-6)	1	202	8.03	0.0051				
				Bird ID (Location)	0.2079 (0.0674)	Moran’s I		2	193	39.48	<0.0001	1 vs. 2	−0.6955 (0.0783)	−8.89	<0.0001
												1 vs. 3	−0.0980 (0.2435)	−0.40	0.6879
												2 vs. 3	0.5976 (0.2521)	2.37	0.0188
4	LMM	210	ln(Foraging distance)	Bird ID	0.3391 (0.1094)	Research area		2	181	9.80	<0.0001	NPHK vs. Bosland	1.1061 (0.2838)	3.90	0.0004
												NPHK vs. Meeuwen	1.7798 (0.4943)	3.60	0.0012
												Bosland vs. Meeuwen	0.6738 (0.4489)	1.50	0.2928
5	LMM	210	sqrt(Foraging habitat)	Bird ID	0.0042 (0.0014)	Research area		2	181	38.98	<0.0001	NPHK vs. Bosland	−0.2812 (0.0319)	−8.82	<0.0001
												NPHK vs. Meeuwen	−0.2342 (0.0554)	−4.23	0.0001
												Bosland vs. Meeuwen	0.0471 (0.0503)	0.94	0.6185
6	LMM	160	ln(Foraging time)	Location	0.0332 (0.1069)	Foraging distance	0.000217 (0.000069)	1	72.4	9.92	0.0024				
				Bird ID (Location)	0.3036 (0.1633)										
7	LMM	199	Flight speed	Bird ID	44.997 (20.735)	Habitat type		1	174	35.04	<0.0001	Suitable vs. Unsuitable	−13.2252 (2.2342)	−5.92	<0.0001
8	LMM	448	ln(Insect biomass)	Trap ID	0.0340 (0.0389)	Temperature	0.1491 (0.0156)	1	439	91.57	<0.0001				
						Functional habitat		3	439	8.27	<0.0001	Inaccessible vs. Breeding type 1	0.8149 (0.2408)	3.38	0.0043
												Inacessible vs. Breeding type 2	0.5375 (0.2405)	2.24	0.1154
												Inacessiblevs. Foraging	−0.2258 (0.2393)	0.94	0.7813
												Breeding type 1 vs. Breeding type 2	−0.2774 (0.2348)	−1.18	0.6391
												Breeding type 1 vs. Foraging	−1.0407 (0.2337)	−4.45	<0.0001
												Breeding type 2 vs. Foraging	−0,7633 (0.2333)	−3.27	0.0063
9	ANOVA	58	Thiol concentration			Research area		2	55	4.82	0.0117	NPHK vs. Bosland	44.892 (14.645)	3.07	0.0093
												NPHK vs. Meeuwen	33.597 (17.465)	1.92	0.1417
												Bosland vs. Meeuwen	−11.296 (16.483)	−0.69	0.7730

### Landscape heterogeneity

Habitat composition is significantly different between the three research areas (df_N_ = 2, df_D_ = 181, F = 38.98, p < 0.0001), with the availability of foraging habitat in proximity to breeding/roosting sites (expressed in average cover percentage) being significantly lower in NPHK (mean = 3 ± 4.5%, original scale) compared to Bosland (19.6 ± 6.7%; t = −8.82, p < 0.0001) and Meeuwen-Gruitrode (14.7 ± 1.3%; −4.23, p = 0.0001; Fig. 2). Foraging distance was modelled via two models. In the first model, it was shown that foraging distance increased when the amount of potential foraging habitat in proximity to breeding/roosting sites decreased (i.e. foraging habitat measured as available area of average foraging distance $\widehat{{\beta }_{1}}=-2.54$, df_N_ = 1, df_D_ = 203, F = 22.58, p < 0.0001) and when habitat diversity increased ($\widehat{{\beta }_{2}}=1.71$, df_N_ = 1, df_D_ = 198, F = 301.54, p < 0.0001). In an alternative version of the first model, it could not be shown that foraging distance was affected by foraging habitat when defining the latter at another scale (i.e. foraging habitat measured as available area of initial foraging distance; $\widehat{{\beta }_{1}}=-0.09$, df_N_ = 1, df_D_ = 197, F = 0.10, p = 0.7471).

From the second model, it was found that foraging distance also increased when landscapes were clustered (df_N_ = 2, df_D_ = 193, F = 39.48, p < 0.0001), and when the size of potential foraging patches increased ($\widehat{{\beta }_{1}}=0.000012$, df_N_ = 1, df_D_ = 202, F = 8.03, p = 0.0051). For the former effect, Tukey-adjusted pairwise comparisons pointed towards significant differences between mixed and clustered landscapes (${\mu }_{diff}=\,-0.70$, df = 203, t = −8.89, p < 0.0001) and clustered and perfectly mixed landscapes (${\mu }_{diff}=0.60$, df = 186, t = 2.37, p = 0.0188). Besides, it is important to note that for both models no other individual (sex, age, wing length) or environmental variables (maximal daily temperature, rain, moon phase) were found to influence nightjars’ foraging distance. Furthermore, it was necessary to correct for individual variation in all models, while variation within locations was shown to be negligible (Table 1).

### Foraging ecology

**Figure 4:**
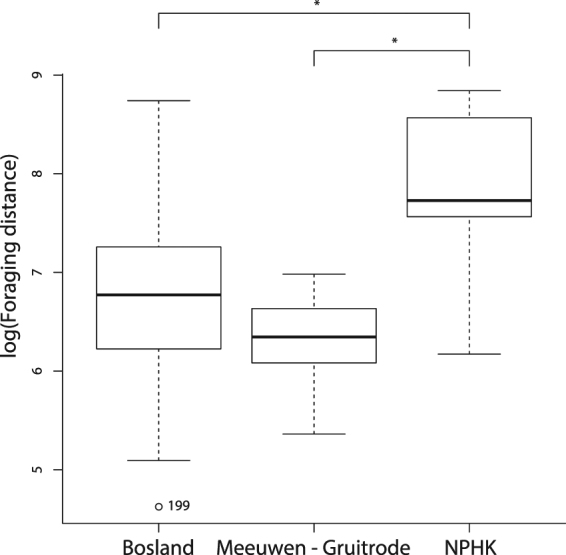
Foraging distances in the Mechelse Heide (National Park Hoge Kempen, NPHK) (3345 ± 1921m, original scale) are significantly longer compared with those in Bosland (1201 ± 1059 m; t = 3.90, p = 0.0004) and in the military area of Meeuwen-Gruitrode (593 ± 271 m; t = 3.60, p = 0.0012). Boxplots show log-transformed foraging distances with median (thick black line), 25% and 75% quantiles (thin box), 90% range (whiskers) and outliers. P-values are based on post-hoc (LMM) Tukey-corrected pairwise t tests (Table 1, Model 4).

**Figure 5:**
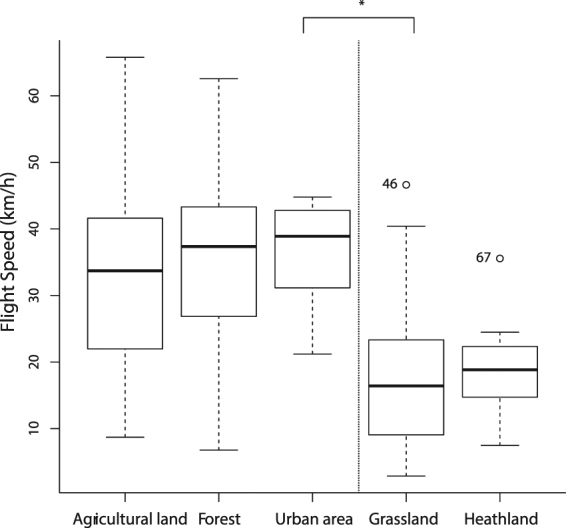
Flight speed (km/h) is significantly higher over unsuitable habitats (35 ± 12 km/h; forest, agricultural land, urban area) compared with more suitable habitats (18 ± 10 km/h; grassland, heathland) (df_N_ = 1, df_D_ = 174, F = 35.04, p < 0.0001). Boxplots show flight speeds (km/h) with median (thick black line), 25% and 75% quantiles (thin box), 90% range (whiskers) and outliers. P-value is based on LMM results (Table 1, Model 7).

We found that foraging distance significantly differed between research areas (df_N_ = 2, df_D_ = 181, F = 9.80, p < 0.0001). Foraging distances were significantly higher in NPHK (3345 ± 1921m; original scale) compared with Bosland (1201 ± 1059 m; t = 3.90, p = 0.0004) and Meeuwen-Gruitrode (593 ± 271 m; t = 3.60, p = 0.0012) (Fig. 4). With increasing foraging distance, nightjars also foraged longer ($\widehat{{\beta }_{1}}=0.000217$, df_N_ = 1, df_D_ = 72.4, F = 9.92, p = 0.0024). We found average flight speeds of 31 ± 13 km/h. However, flight speed significantly differed between habitats (df_N_ = 1, df_D_ = 174, F = 35.04, p < 0.0001). Flight speeds were higher over unsuitable habitats (35 ± 12 km/h) compared with passage through potential foraging and breeding habitats (18 ± 10 km/h) (Fig. 5).

### Foraging economy

We collected 18911 individuals of *Lepidoptera*. Moth biomass was higher when temperatures increased ($\widehat{{\beta }_{1}}=0.1491$, df_N_ = 1, df_D_ = 439, F = 91.57, p < 0.0001), while it also varied between habitats (df_N_ = 3, df_D_ = 439, F = 8.27, p < 0.0001). Moth biomass was significantly higher in foraging habitats when compared with breeding habitats, but not when compared with observations in pine forest.

**Figure 6:**
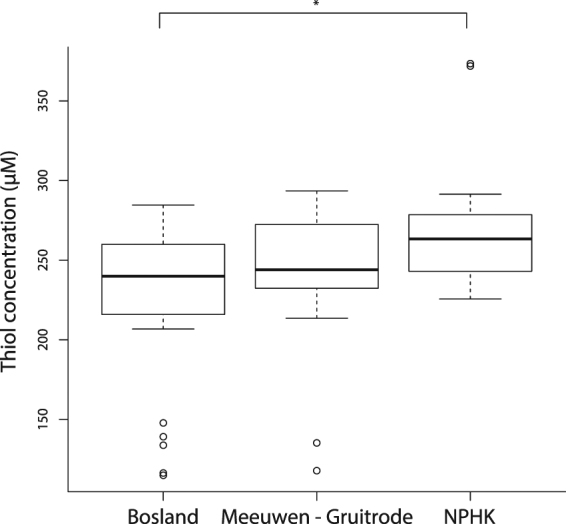
Plasma concentrations of thiols are significantly higher in birds in the Mechelse Heide (National Park Hoge Kempen, NPHK) (268.67 µmol/L ± 41.64; t = 3.07, p = 0.0093) when compared with those in Bosland (223.78 µmol/L ± 50.32), but not when compared with those in the military area of Meeuwen-Gruitrode (235.08 µmol/L ± 54; t = 1.92, p = 0.1417). Boxplots show thiol concentrations (µM) with median (thick black line), 25% and 75% quantiles (thin box), 90% range (whiskers) and outliers. P-values are based on post-hoc (ANOVA) Tukey-corrected pairwise t tests (Table 1, Model 9).

When comparing plasma concentration of thiols for each bird between research areas, a significant overall effect was found (df_N_ = 2, df_D_ = 55, F = 4.82, p = 0.0117), with plasma concentrations for birds of the NPHK (268.67µmol/L ± 41.64*; t* = *3*.07, *p* = *0*.*0093)* being significantly higher when compared with those of birds in Bosland *(223*.*78* *µmol/L* ± *50*.*32)*, but not when compared with birds in Meeuwen-Gruitrode *(235*.*08* *µmol/L* ± *54; t* = *1*.92, *p* = *0*.*1417;* Fig. 6).

## Discussion

The use of GPS-loggers yielded detailed information on the movement of 30 nightjars within their respective home ranges. Tracking data allowed us to study foraging behaviour and actual movement paths of 210 foraging flights collected in three research areas. Our findings indicate that not only landscape composition, as already indicated by Evens *et al*.^5^, but also landscape configuration influences the connectivity between two complementary resources for nightjars: nest sites and foraging areas. On a daily basis, nightjars connect breeding and foraging sites by rapidly crossing unsuitable habitats in order to exploit a higher prey biomass in foraging sites. Increased stress levels were found in birds occupying large, attractive, yet sub-optimal breeding areas. These birds travel longer distances over unsuitable habitat and compensate for longer travel time by spending more time in foraging habitats (i.e. longer foraging time).

### Landscape heterogeneity

Two measures of habitat composition influence nightjars’ foraging distance in our case: the availability of potential foraging habitat and the habitat diversity. The amount of unsuitable habitat that separates breeding and foraging habitats differs between our study sites, which could play a role in altering foraging distances. In NPHK, for example, large pine forests surround the heathlands, reducing the availability of potential foraging habitat in the vicinity of nightjars’ nesting sites (i.e. foraging habitat measured as available area of average foraging distance). Consequently, nightjars from NPHK are obliged to forage three to six times further away from their nesting sites than are those from Bosland or Meeuwen-Gruitrode. On a wider scale (i.e. foraging habitat measured as available area of initial foraging distance), however, the availability of potential foraging habitat does not influence nightjars’ foraging distance. On the one hand, this could mean that habitat measurements at a larger spatial scale become irrelevant in relation to nightjars’ foraging distance and that only habitat characteristics on a small spatial scale are useful to predict their foraging behaviour. On the other hand, our findings could indicate that nightjars require daily access to a specific amount of foraging habitat, for which some have to travel further comparted to others, due to their initial choice of breeding site. Evens *et al*.^5^ already indicated differences in foraging distance between telemetry studies in the UK and Belgium. Our results suggest that this observation could be possibly explained by variation in landscape heterogeneity between study areas.

In degraded, homogenized landscapes, birds generally fly further to find food compared to those in diverse landscapes^6,28,43,44^. However, in our study we found that nightjars’ foraging distance is longer in diverse landscapes. This can be explained by the high diversity in unsuitable and roosting habitat types in our specific case, and by the low diversity in foraging habitat types. Nightjars generally forage near specific landscape elements, such as scattered trees or hedgerows in extensively-grazed grasslands, ponds, oak shrubland and wet meadows^5,38,45^. These foraging habitats usually are relics in vast agricultural lands or pine forests, where landscape fragmentation and homogenisation is still ongoing. Higher food abundance in larger patches^46^ could explain why nightjars fly further to find larger foraging habitats in our study. As such, ongoing homogenisation of landscapes and fragmentation of foraging habitats will continue to reduce food resources because fragmented foraging habitats and specific landscape elements are being further eliminated^43^, which will increase the foraging distance for nightjars.

Besides habitat composition, habitat configuration also affects nightjars’ foraging behaviour. Foraging distance is three times greater when functional habitats are clustered. In clustered landscapes, longer foraging distances can be explained by the nightjars having to cross larger distances across unsuitable habitat. In randomly-distributed landscapes, breeding and foraging habitats can be found on a much smaller spatial scale^9^. This implies that, when the availability of foraging habitat and habitat diversity is held constant, modification of landscape configuration also influences the connectivity between functional habitats^14^ and can affect individual^17^, population^47^ and ecosystem processes^48^.

### Foraging ecology

Being highly mobile, nightjars show the ability to connect complementary habitats that are separated by 100 m up to seven kilometres. We observed that flight speed is habitat dependant, as nightjars cross agricultural land, forests and urban areas faster than they cross grasslands and heathlands. Generally, animals move faster between resources when the expected food availability in target patches is higher^49^, or when moving through less-preferred habitats^50,51^, especially when movement through these habitats is risky^52,53^ or if resources are low^52,54^. We found that in the foraging habitats and pine forests of Bosland moth biomasses are two to three times higher than they are in the breeding habitats. In pine forests, however, nightjars do not forage because of visual limitations^38^. Further assuming that food availability is distributed evenly in similar foraging habitats, it seem likely that nightjars increase flight speed as a response to lower food resources in agricultural land and above dense forests. Opportunistic, supplementary feeding on-the-wing might explain the reduced flight speeds across grasslands and heathlands.

### Foraging economy

We found that nightjars prolong foraging time when foraging distance increase. Therefore, nightjars probably attempt to balance the costs of travelling against the benefits of energy acquisition to maximise net-energy gain (sensu Hedenstrom and Alerstam^55^). Variation in landscape heterogeneity thus can have a profound impact on nightjars’ daily energy expenditure as greater distances across unsuitable habitats are harder to cross^56^ and higher flight speeds are also more energetically demanding^55^.

The allocation of more time and energy for foraging most likely also influences the fitness of nightjars through reduced reproductive success^6,16,20,28,57^ and increased predation risk^29^. We found that plasma concentration of thiols, indicator for plasma antioxidant levels^58^, is significantly higher in birds from NPHK compared with those from Bosland. Nightjars from NPHK forage further and travel longer distances across unsuitable habitats, which is more energy demanding and may have led to elevated levels of oxidative stress. Birds occupying sub-optimal breeding habitats thus may experience a lower fitness as shown by the elevated antioxidant defences^31,32,59^.

We can expect that reduced food availability^30,60^ and food quality^61,62^ also affects nightjars’ oxidative status. Despite having shorter foraging distances, nightjars from Meeuwen-Gruitrode show intermediate levels of thiol concentrations. In contrast to the large, extensively-cultivated grasslands in Bosland and NPHK, foraging sites in Meeuwen-Gruitrode consist of small landscape elements in intensively-cultivated farmlands that possibly hold lower quality food^63^. Evidently, further work is needed to fully understand the role of plasma thiols in the way they are influenced by environmental stressors^58^ and how they relate to survival and reproductive success in nightjars.

## Conclusion

Our study shows that landscape heterogeneity can affect the connectivity between nightjars’ functional habitats, influence their foraging behaviour and might also affect individuals’ health and population processes. However, current conservation plans for nightjars, developed within the Natura 2000 framework, focus on the management of heathlands (i.e. breeding habitat)^23^ and ignore the importance of key foraging habitats. Following our results we, therefore, conclude that (1) Natura 2000 objectives should be revised, (2) creation of new breeding grounds should be preceded by an assessment of landscape heterogeneity to minimize the distance between breeding and foraging sites and (3) restoration of known breeding grounds should also focus on creating/restoring foraging habitats in proximity to these breeding grounds.

### Experiments on live vertebrates

The authors declare that all experiments have been performed under licenses of the Royal Belgian Institute for Natural Sciences (bird ringing licence) and the Flemish Agency Nature and Forest (GPS-tagging, blood sampling with Felasa B licence).

## Electronic supplementary material

Supplementary information
