# Author Correction: Proximity of breeding and foraging areas affects foraging effort of a crepuscular, insectivorous bird

**DOI:** 10.1038/s41598-018-25897-5

**Published:** 2018-06-20

**Authors:** Ruben Evens, Natalie Beenaerts, Thomas Neyens, Nele Witters, Karen Smeets, Tom Artois

**Affiliations:** 1 Hasselt University, Centre for Environmental Sciences, Research Group: Zoology, Biodiversity and Toxicology, Campus Diepenbeek, Agoralaan, Gebouw D, 3590 Diepenbeek, Belgium; 2 Hasselt University, Centre for Statistics, Research Group: I-BIOSTAT, Campus Diepenbeek, Agoralaan, Gebouw D, 3590 Diepenbeek, Belgium; 3 Hasselt University, Centre for Environmental Sciences, Research Group: Environmental Economics, Campus Diepenbeek, Agoralaan, Gebouw D, 3590 Diepenbeek, Belgium

Correction to: *Scientific Reports* 10.1038/s41598-018-21321-0, published online 14 February 2018

The Acknowledgements section in this Article is incomplete.

“RE is funded by a BOF-mandate (BSFFCMKDK) at Hasselt University. NW is funded as a postdoctoral researcher by FWO Flanders. Part of this research was funded by another FWO-grant (N1522015). We thank Eddy Ulenaers, Luc Lens, Dries Gorissen, Guido Winters, Koen Thijs, Lise Hendrick, Karen Vanmarcke, Fien Evens, Albert Geuens, Lyndon Kearsley, Greg Conway, Ian Henderson and Nikki Watson for their support and relevant comments on this manuscript. Research equipment was funded by the Agency for Nature and Forest (ANB; Belgium). Permissions were granted by the ANB and Royal Belgian Institute of Natural Sciences (Belgium).”

should read:

“RE is funded by a BOF-mandate (BSFFCMKDK) at Hasselt University. NW and TN are funded as postdoctoral researchers by FWO Flanders. Part of this research was funded by another FWO-grant (N1522015). We thank Eddy Ulenaers, Luc Lens, Dries Gorissen, Guido Winters, Koen Thijs, Lise Hendrick, Karen Vanmarcke, Fien Evens, Albert Geuens, Lyndon Kearsley, Greg Conway, Ian Henderson and Nikki Watson for their support and relevant comments on this manuscript. Research equipment was funded by the Agency for Nature and Forest (ANB; Belgium). Permissions were granted by the ANB and Royal Belgian Institute of Natural Sciences (Belgium).”

