# Supplementary material for: Proximity of breeding and foraging areas affects foraging effort of a crepuscular, insectivorous bird
Source: Sci Rep. 2018 Feb 14;8:3008. doi: 10.1038/s41598-018-21321-0 (PMC5813100; doi:10.1038/s41598-018-21321-0)
Supplement: Supplementary file 1 — Supplementary information [file 41598_2018_21321_MOESM1_ESM.doc]

**Proximity of breeding and foraging areas affects foraging effort of a crepuscular, insectivorous bird**

Ruben Evens^1^*, Natalie Beenaerts^1^, Thomas Neyens^2^, Nele Witters³, Karen Smeets^1^ & Tom Artois^1^

^1^ Hasselt University, Centre for Environmental Sciences, Research Group: Zoology, Biodiversity and Toxicology, Campus Diepenbeek, Agoralaan, Gebouw D, 3590 Diepenbeek, Belgium

^2^Hasselt University, Centre for Statistics, Research Group: I-BIOSTAT, Campus Diepenbeek, Agoralaan, Gebouw D, 3590 Diepenbeek, Belgium

^3^Hasselt University, Centre for Environmental Sciences, Research Group: Environmental Economics, Campus Diepenbeek, Agoralaan, Gebouw D, 3590 Diepenbeek, Belgium

* Corresponding author: [ruben.evens@uhasselt.be](mailto:ruben.evens@uhasselt.be), 0032/11268291

**Supplementary Methods M1: Calculation of foraging distance, flight speed and foraging time.**

We followed seven steps to calculate foraging distance, flight speed and foraging times. Tracking data of each bird was analysed similarly. As an example, we here explain how we processed the data collected for one bird.

1. We separated observations from subsequent nights (i.e. data from dusk till dawn) (Supplementary Figure 2a).
2. We defined *complete foraging tracks* as movements that include the start at breeding/roosting site, flight towards foraging habitats, foraging, return flight to breeding site and arrival at breeding/roosting site (Supplementary Figure 2b). It is possible that multiple foraging *tracks* occurred during one night.
3. For each complete or incomplete *track*, we separated observations that indicated stationary or flying behaviour (Supplementary Figure 2b).
   1. Stationary periods could be recognised as clustered observations in breeding or foraging sites.
   2. Flight paths could be recognised as linear observations that indicate directional movement. In order to visualise flight paths, we selected the last stationary observation at the departure site, all observations that indicated linear flight and c) the first stationary observation in the arrival site.
4. We calculated departure time and foraging duration (i.e. time spent stationary in foraging habitats) for each *complete foraging track*.
5. We separated all flight paths within a *track* (Supplementary Figure 2c). For each flight path, we calculated the effective length (cumulative distance between every observation) (QGIS: field calculator).
6. We defined *initial foraging flights* as a measure of foraging distance (Supplementary Figure 2c). Initial foraging flights were identified as the first flight per night between breeding/roosting site and foraging habitat, and the subsequent foraging flights with at least one hour intervals.
7. We calculated habitat-specific flight speed by superimposing flight paths on a detailed habitat map (Biological Value Map version 2.2; De Saeger et al. 2016). Flight speed was calculated as the distance between two consecutive observations, divided by the time between those two observations (Supplementary Figure 2).


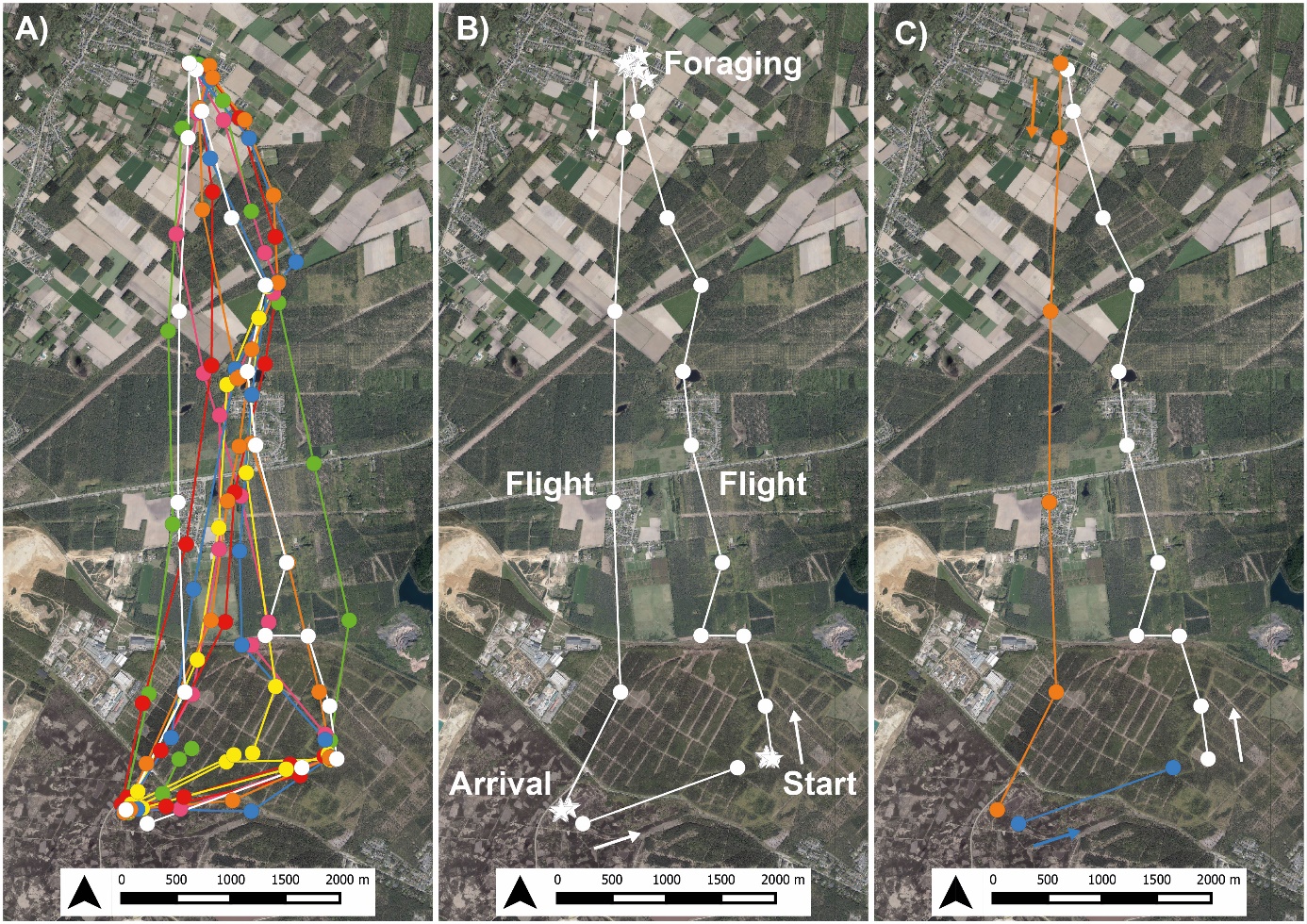


*Supplementary Figure 1: Tracking data of one nightjar collected in NPHK, plotted on an orthophotographic map. A) Tracking data was first separated by night (here seven nights). Every colour represents one night (bullets = observations, lines connect subsequent observations). B) In this particular night, we observed one complete foraging track. This complete foraging track consists of a start at the roosting site, flight towards foraging site, foraging, return flight and arrival at territory. At dawn there was an additional flight from territory (arrival) towards roosting site (start). Arrows = flight direction, stars = stationary behaviour, bullets = movement, lines connect subsequent observations. C) The foraging track was subsequently divided into three flight paths: initial foraging track (white), return flight towards territory (orange), return flight towards roost (blue). The background map was used under open data access of Google Maps; maps were created using QGIS 2.12 Lyon* (Open Source Geospatial Foundation Project, [http://qgis.osgeo.org](http://qgis.osgeo.org/)) *and edited using Adobe Illustrator CC www.adobe.com.*


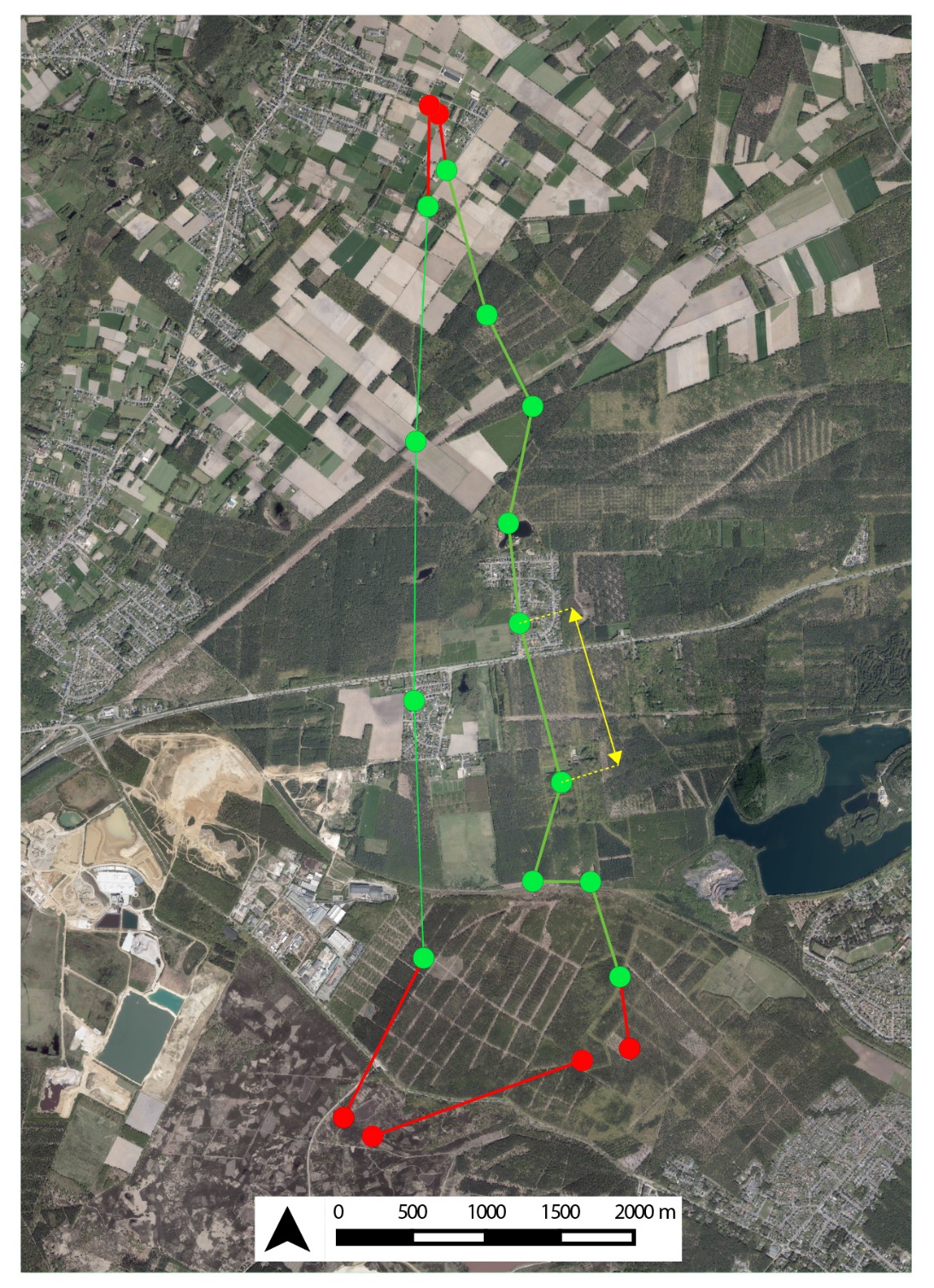


*Supplementary Figure 2: Calculation of flight speed. This figure shows the same observations as Supplementary Figure 1c (i.e. GPS-observations on an orthophotographic map). Red = stationary points which were used to visualise flight paths, green = flight observations which were used to calculate flight speed, yellow arrow = an example of how flight speed was calculated as distance divided by the time between flight observations. The background map was used under open data access of Google Maps; maps were created using QGIS 2.12 Lyon* (Open Source Geospatial Foundation Project, [http://qgis.osgeo.org](http://qgis.osgeo.org/)) *and edited using Adobe Illustrator CC www.adobe.com.*

**Supplementary Methods M2: Creation of sub-maps.**

For each initial foraging flight (210 in total) we created four sub-maps as circular cuts from the structural and the functional habitat map, to delineate the total surface area of available habitats and derive measures of local landscape heterogeneity, based on Evens et al. 2017. Below we show in detail how those sub-maps were produced for one initial foraging flight.

First, as discussed in the main text, we started by developing tailored structural (Supplementary Figure 3, left) and functional maps (Supplementary Figure 3, right).


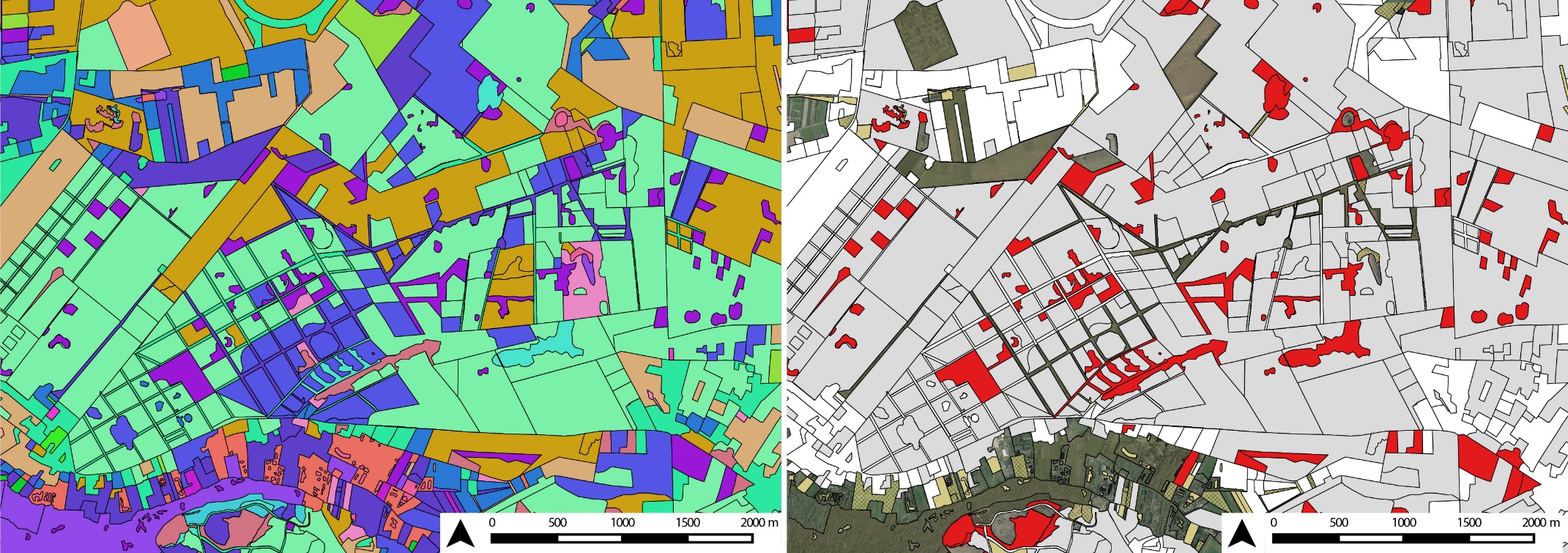


*Supplementary Figure 3: Tailored structural (left) and functional map (right). Structural map: every colour represents one out of 23 habitat types. Functional map: red = breeding habitat, grey = roosting habitat, white = unsuitable habitat, greenish/yellow = foraging habitat. The habitat maps were modified from the Biological Value Map V2.0 under open data access of INBO; maps were created using QGIS 2.12 Lyon* (Open Source Geospatial Foundation Project, [http://qgis.osgeo.org](http://qgis.osgeo.org/)) *and edited using Adobe Illustrator CC www.adobe.com.*

Next, for each initial foraging flight we created two circular surfaces which represent the available habitat for that initial foraging flight. We plotted both circles on the structural and functional map (Supplementary Figure 4).The radius of the first circle equals the length of the foraging flight. The radius of the second circle equals the average foraging distance (1650m). The average foraging distance was calculated as the average foraging distance of all initial foraging flights for all individuals. Centre of the circles is the start location of that initial foraging flight.


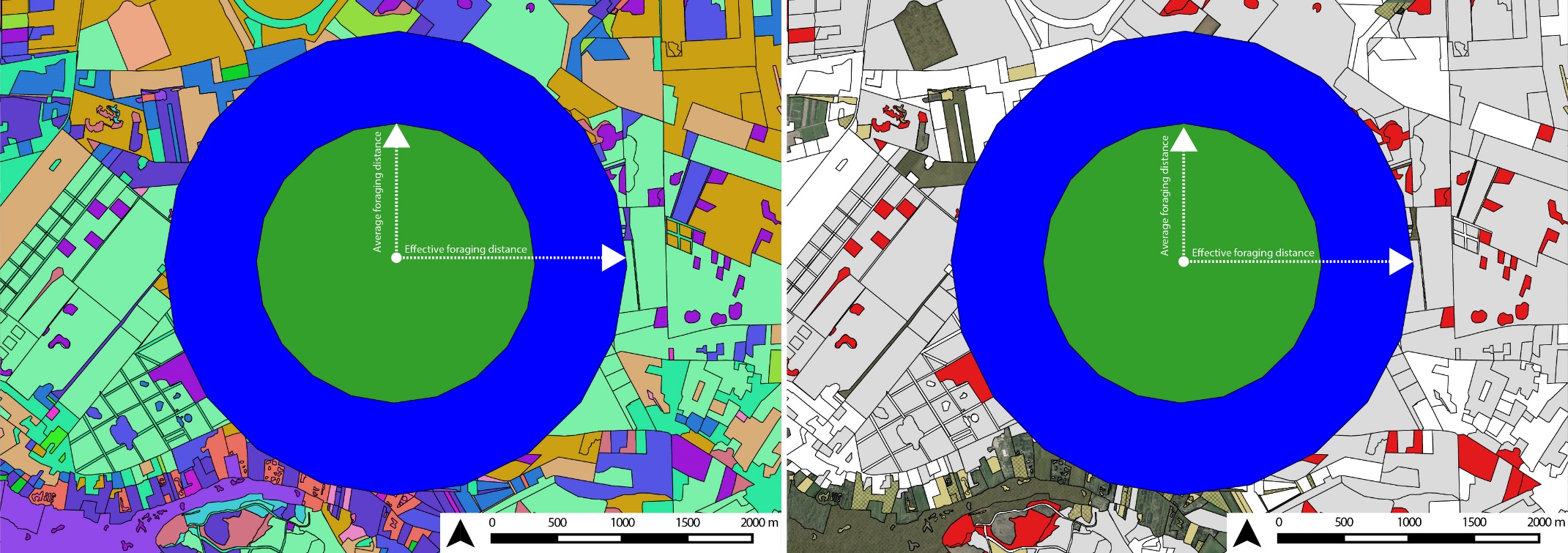


*Supplementary Figure 4: Plotting circles of available habitat on the structural and functional map. Tailored structural (left) and functional map (right). Structural map: every colour represents one out of 23 habitat types. Functional map: red = breeding habitat, grey = roosting habitat, white = unsuitable habitat, greenish/yellow = foraging habitat. Blue circle = available habitat for effective foraging distance, green = available habitat for average foraging distance, centre = start of initial foraging flight. The habitat maps were modified from the Biological Value Map V2.0 under open data access of INBO; maps were created using QGIS 2.12 Lyon* (Open Source Geospatial Foundation Project, [http://qgis.osgeo.org](http://qgis.osgeo.org/)) *and edited using Adobe Illustrator CC www.adobe.com.*

Finally, we cut out the circles from the structural and functional habitat maps to produce sub-maps of available habitat. In supplementary figure 5 we show two – out of four – sub-maps that were made for this initial foraging flight.


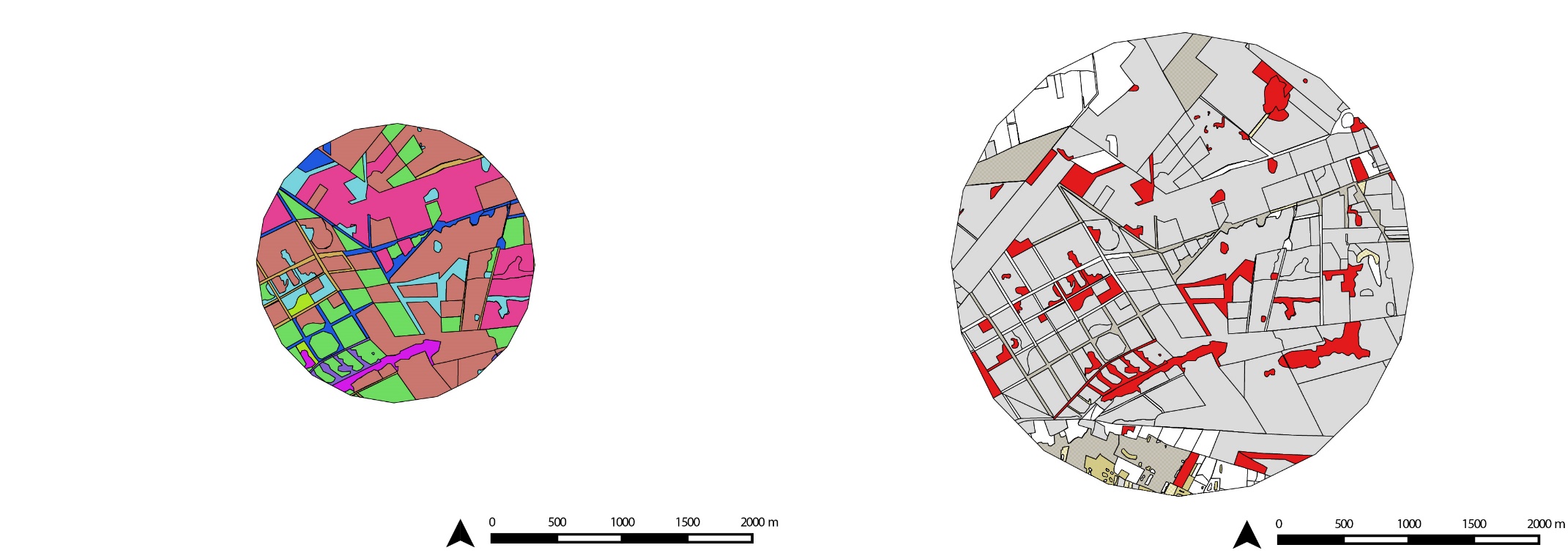


*Supplementary Figure 5: Two out of four sub-maps from structural and functional habitat maps created for one initial foraging flight. Left: sub-map structural habitat for median foraging distance. Every colour represents one out of 23 habitat types, radius = 1650m. Right: sub-map functional habitat for effective foraging distance. Functional map: red = breeding habitat, grey = roosting habitat, white = unsuitable habitat, darker grey/greenish = foraging habitat, radius = effective foraging distance. The habitat maps were modified from the Biological Value Map V2.0 under open data access of INBO; maps were created using QGIS 2.12 Lyon* (Open Source Geospatial Foundation Project, [http://qgis.osgeo.org](http://qgis.osgeo.org/)) *and edited using Adobe Illustrator CC www.adobe.com.*

**Supplementary Table T1: Overview of tagged nightjars.**

We tagged 48 nightjars for the purpose of this study and we recaptured 31. Below, we show more details regarding the deployments. Table 1: ID = ring number, Sex = M (Male) or F (Female), Age = CY (Calendar Year), Location = NPHK (National Park Hoge Kempen), Weight (g), Wing = Wing length (mm), TW = Tag Weight (g), SF = Sampling Frequency (min), Obs = number of observations collected.

| ID | Year | Date | Sex | Age | Location | Latitude | Longitude | Weight | Wing | Manufacturer | Tag type | TW | SF | Obs |
| --- | --- | --- | --- | --- | --- | --- | --- | --- | --- | --- | --- | --- | --- | --- |
| 21Z03987 | 2015 | 07/08/2015 | M | >2CY | Bosland | 51.172922 | 5.333318 | 71.6 | 189 | Biotrack Ltd. | PinPoint-40 V2 | 2 | 4 | 364 |
| 21Z03991 | 2016 | 23/06/2016 | F | >2 CY | Bosland | 51.165644 | 5.313132 | 80.3 | 195 | Pathtrack Ltd. | nanoFix V2 | 2 | 4 | 388 |
| 21Z39198 | 2015 | 24/06/2015 | M | >2 CY | Bosland | 51.166121 | 5.331603 | 60.3 | 192 | Pathtrack Ltd. | nanoFix V1 | 2 | 3 | 0 |
| 22Z70320 | 2015 | 24/06/2015 | M | >1 CY | Bosland | 51.166697 | 5.33381 | 63.8 | 189 | Biotrack Ltd. | PinPoint-40 V2 | 2 | 4 | 444 |
| 22Z70330 | 2015 | 19/06/2015 | M | >2 CY | Bosland | 51.183243 | 5.345704 | 66.3 | 201 | Biotrack Ltd. | PinPoint-40 V2 | 2 | 4 | 439 |
| 22Z70342 | 2014 | 23/06/2014 | M | >2 CY | Meeuwen-Gruitrode | 51.066457 | 5.510024 | 71.9 | 194 | Biotrack Ltd. | PinPoint-40 V1 | 2 | 15 | 40 |
| 22Z70342 | 2016 | 07/06/2016 | M | >2 CY | Meeuwen-Gruitrode | 51.066457 | 5.510024 | 71.9 | 194 | Pathtrack Ltd. | nanoFix V2 | 2.5 | 3 | 0 |
| 22Z70395 | 2016 | 30/05/2016 | M | >2 CY | Bosland | 51.142067 | 5.34199 | 64.9 | 188 | Pathtrack Ltd. | nanoFix V2 | 2.5 | 3 | 0 |
| 23Z32605 | 2014 | 15/06/2014 | F | >2 CY | Bosland | 51.139911 | 5.343266 | 87 | 192 | Biotrack Ltd. | PinPoint-40 V1 | 2 | 15 | 40 |
| 23Z32606 | 2014 | 15/06/2014 | F | 2 CY | Bosland | 51.13941 | 5.34242 | 83 | 202 | Biotrack Ltd. | PinPoint-40 V1 | 2 | 15 | 40 |
| 23Z32608 | 2014 | 18/06/2014 | F | 2 CY | Bosland | 51.178004 | 5.240219 | - | - | Biotrack Ltd. | PinPoint-40 V1 | 2 | 15 | 0 |
| 23Z32610 | 2014 | 20/06/2014 | F | >2 CY | Meeuwen-Gruitrode | 51.064721 | 5.499867 | 89.3 | 192 | Biotrack Ltd. | PinPoint-40 V1 | 2 | 15 | 0 |
| 23Z32617 | 2014 | 25/06/2014 | F | >2 CY | Bosland | 51.183376 | 5.303594 | - | 203 | Biotrack Ltd. | PinPoint-40 V1 | 2 | 15 | 40 |
| 23Z32623 | 2015 | 23/05/2015 | F | >2 CY | Bosland | 51.18309 | 5.303946 | 71.3 | 195 | Biotrack Ltd. | PinPoint-40 V2 | 2 | 4 | 224 |
| 23Z32623 | 2016 | 12/07/2016 | F | >2 CY | Bosland | 51.18325 | 5.302453 | 71.3 | 195 | Pathtrack Ltd. | nanoFix V2 | 2 | 4 | 431 |
| 23Z32624 | 2015 | 09/06/2015 | M | >2 CY | Bosland | 51.18309 | 5.303946 | 67.7 | 194 | Pathtrack Ltd. | nanoFix V1 | 2 | 3 | 307 |
| 23Z32639 | 2015 | 24/06/2015 | F | >1 CY | Bosland | 51.166121 | 5.331603 | 68.2 | 197 | Biotrack Ltd. | PinPoint-40 V2 | 2 | 3 | 447 |
| 23Z32666 | 2016 | 07/06/2016 | M | >2 CY | Meeuwen-Gruitrode | 51.065863 | 5.510087 | 74 | 194 | Pathtrack Ltd. | nanoFix V2 | 2 | 3 | 0 |
| 23Z32671 | 2016 | 24/05/2016 | F | >2 CY | Bosland | 51.172548 | 5.297242 | 78 | 196 | Pathtrack Ltd. | nanoFix V2 | 2 | 4 | 837 |
| 23Z32673 | 2016 | 24/05/2016 | M | >2 CY | Bosland | 51.196635 | 5.290765 | 79.2 | 192 | Pathtrack Ltd. | nanoFix V2 | 2.5 | 3 | 1050 |
| 23Z32681 | 2015 | 10/06/2015 | F | >2 CY | Meeuwen-Gruitrode | 51.066633 | 5.510557 | 63.4 | 187 | Pathtrack Ltd. | nanoFix V1 | 2 | 3 | 567 |
| 23Z32681 | 2016 | 17/05/2016 | F | >2 CY | Meeuwen-Gruitrode | 51.066633 | 5.510557 | 63.4 | 187 | Pathtrack Ltd. | nanoFix V2 | 2 | 4 | 500 |
| 23Z32684 | 2015 | 16/06/2015 | M | 2 CY | Bosland | 51.178004 | 5.240219 | 58.4 | 189 | Biotrack Ltd. | PinPoint-40 V2 | 2 | 4 | 537 |
| 23Z32684 | 2016 | 25/05/2016 | M | >2 CY | Bosland | 51.178004 | 5.240219 | 58.4 | 189 | Pathtrack Ltd. | nanoFix V2 | 2.5 | 3 | 0 |
| 23Z32687 | 2015 | 18/06/2015 | M | > 1CY | NPHK | 50.977304 | 5.642359 | 68.5 | 190 | Biotrack Ltd. | PinPoint-40 V2 | 2 | 4 | 452 |
| 23Z32688 | 2015 | 18/06/2015 | M | >1 CY | NPHK | 50.977304 | 5.642359 | 65.4 | 193 | Biotrack Ltd. | PinPoint-40 V2 | 2 | 4 | 498 |
| 23Z32691 | 2015 | 19/06/2015 | F | >1 CY | Bosland | 51.166121 | 5.331603 | 77.9 | 194 | Pathtrack Ltd. | nanoFix V1 | 2 | 3 | 0 |
| 23Z32699 | 2015 | 25/06/2015 | F | 2 CY | NPHK | 50.977304 | 5.642359 | 83.6 | 196 | Biotrack Ltd. | PinPoint-40 V2 | 2 | 4 | 0 |
| 23Z85683 | 2016 | 10/05/2016 | M | >2 CY | Bosland | 51.19501 | 5.285976 | 79.7 | 195 | Pathtrack Ltd. | nanoFix V2 | 2.5 | 3 | 1072 |
| 23Z85701 | 2015 | 30/06/2015 | M | >2 CY | Meeuwen-Gruitrode | 51.063884 | 5.511421 | 66.5 | 192 | Pathtrack Ltd. | nanoFix V1 | 2 | 3 | 432 |
| 23Z85704 | 2015 | 30/06/2015 | F | 2 CY | Meeuwen-Gruitrode | 51.063884 | 5.511421 | - | 194 | Pathtrack Ltd. | nanoFix V1 | 2 | 3 | 0 |
| 23Z85706 | 2016 | 17/05/2016 | M | >2 CY | NPHK | 50.981514 | 5.627602 | 70.05 | 194 | Pathtrack Ltd. | nanoFix V2 | 2 | 3 | 0 |
| 23Z85710 | 2015 | 09/07/2015 | F | >2 CY | NPHK | 50.980714 | 5.627992 | 75.1 | 191 | Pathtrack Ltd. | nanoFix V1 | 2 | 3 | 0 |
| 23Z85720 | 2016 | 16/05/2016 | M | >2 CY | Bosland | 51.183533 | 5.302382 | 69 | 187 | Pathtrack Ltd. | nanoFix V2 | 2 | 3 | 504 |
| 23Z85727 | 2015 | 23/07/2015 | F | 2 CY | Bosland | 51.192548 | 5.288049 | 65.5 | 195 | Biotrack Ltd. | PinPoint-40 V2 | 2 | 4 | 449 |
| 23Z85729 | 2016 | 18/05/2016 | M | >2 CY | NPHK | 50.981514 | 5.627602 | 67.5 | 196 | Pathtrack Ltd. | nanoFix V2 | 2.5 | 3 | 1145 |
| 23Z85732 | 2016 | 07/06/2016 | M | >2 CY | NPHK | 50.984918 | 5.61607 | 57.2 | - | Pathtrack Ltd. | nanoFix V2 | 2 | 3 | 0 |
| 23Z85734 | 2015 | 28/07/2015 | M | >2 CY | Bosland | 51.171893 | 5.339412 | 61.7 | 192 | Biotrack Ltd. | PinPoint-40 V2 | 2 | 4 | 0 |
| 23Z85806 | 2016 | 10/05/2016 | F | >2 CY | Bosland | 51.19501 | 5.285976 | 97.2 | 197 | Pathtrack Ltd. | nanoFix V2 | 2 | 3 | 490 |
| 23Z85811 | 2016 | 17/05/2016 | F | >2 CY | NPHK | 50.981514 | 5.627602 | 83.2 | 200 | Pathtrack Ltd. | nanoFix V2 | 2 | 3 | 936 |
| 23Z85819 | 2016 | 21/05/2016 | M | >1 CY | Bosland | 51.136811 | 5.341899 | 70 | 193 | Pathtrack Ltd. | nanoFix V2 | 2 | 3 | 818 |
| 23Z85820 | 2016 | 24/05/2016 | M | >2 CY | Bosland | 51.172548 | 5.297242 | 69 | 195 | Pathtrack Ltd. | nanoFix V2 | 2 | 3 | 0 |
| 23Z85824 | 2016 | 10/06/2016 | F | 2 CY | Bosland | 51.19537 | 5.291996 | 74.7 | 201 | Pathtrack Ltd. | nanoFix V2 | 2 | 3 | 717 |
| 23Z85825 | 2016 | 06/06/2016 | M | 2 CY | NPHK | 50.981514 | 5.627602 | - | 191 | Pathtrack Ltd. | nanoFix V2 | 2.5 | 3 | 846 |
| 23Z85826 | 2016 | 07/06/2016 | F | >2 CY | NPHK | 50.984918 | 5.61607 | - | 194 | Pathtrack Ltd. | nanoFix V2 | 2 | 3 | 665 |
| 23Z85831 | 2016 | 15/06/2016 | M | 2 CY | Bosland | 51.140492 | 5.344451 | 75.7 | 194 | Pathtrack Ltd. | nanoFix V2 | 2.5 | 3 | 666 |
| 23Z85840 | 2016 | 27/06/2016 | F | 2 CY | Bosland | 51.167286 | 5.350144 | - | 194 | Pathtrack Ltd. | nanoFix V2 | 2 | 3 | 0 |
| 23Z85846 | 2016 | 07/07/2016 | F | 2 CY | NPHK | 50.984918 | 5.61607 | 72.9 | 194 | Pathtrack Ltd. | nanoFix V2 | 2 | 4 | 0 |
